# Gender-Based Violence Narratives in Internet-Based Conversations in Nigeria: Social Listening Study

**DOI:** 10.2196/46814

**Published:** 2023-09-15

**Authors:** Martha Silva, Udochisom Anaba, Nrupa Jani Tulsani, Pooja Sripad, Jonathan Walker, Adolor Aisiri

**Affiliations:** 1 Department of International Health and Sustainable Development School of Public Health and Tropical Medicine Tulane University New Orleans, LA United States; 2 Population Council Washington, DC United States; 3 Fluency M&C Saatchi New York, NY United States; 4 Department of International Health and Sustainable Development School of Public Health and Tropical Medicine Tulane University Abuja Nigeria

**Keywords:** gender-based violence, social listening, sexual health, consent, social media, Twitter, Nigeria, gender inequalities, discrimination, natural language processing, sexual consent

## Abstract

**Background:**

Overcoming gender inequities is a global priority recognized as essential for improved health and human development. Gender-based violence (GBV) is an extreme manifestation of gender inequities enacted in real-world and internet-based environments. In Nigeria, GBV has come to the forefront of attention since 2020, when a state of emergency was declared due to increased reporting of sexual violence. Understanding GBV-related social narratives is important to design public health interventions.

**Objective:**

We explore how gender-related internet-based conversations in Nigeria specifically related to sexual consent (actively agreeing to sexual behavior), lack of consent, and slut-shaming (stigmatization in the form of insults based on actual or perceived sexuality and behaviors) manifest themselves and whether they changed between 2017 and 2022. Additionally, we explore what role events or social movements have in shaping gender-related narratives in Nigeria.

**Methods:**

Social listening was carried out on 12,031 social media posts (Twitter, Facebook, forums, and blogs) and almost 2 million public searches (Google and Yahoo search engines) between April 2017 and May 2022. The data were analyzed using natural language processing to determine the most salient conversation thematic clusters, qualitatively analyze time trends in discourse, and compare data against selected key events.

**Results:**

Between 2017 and 2022, internet-based conversation about sexual consent increased 72,633%, from an average 3 to 2182 posts per month, while slut-shaming conversation (perpetrating or condemning) shrunk by 9%, from an average 3560 to 3253 posts per month. Thematic analysis shows conversation revolves around the objectification of women, poor comprehension of elements of sexual consent, and advocacy for public education about sexual consent. Additionally, posters created space for sexual empowerment and expressions of sex positivity, pushing back against others who weaponize posts in support of slut-shaming narrative. Time trend analysis shows a greater sense of empowerment in advocating for education around the legal age of consent for sexual activity, calling out double standards, and rejecting slut-shaming. However, analysis of emotions in social media posts shows anger was most prominent in sexual consent (n=1213, 73%) and slut-shaming (n=226, 64%) posts. Organic social movements and key events (#ArewaMeToo and #ChurchToo, the #SexforGrades scandal, and the #BBNaija television program) played a notable role in sparking discourse related to sexual consent and slut-shaming.

**Conclusions:**

Social media narratives are significantly impacted by popular culture events, mass media programs, social movements, and micro influencers speaking out against GBV. Hashtags, media clips, and other content can be leveraged effectively to spread awareness and spark conversation around evolving gender norms. Public health practitioners and other stakeholders including policymakers, researchers, and social advocates should be prepared to capitalize on social media events and discourse to help shape the conversation in support of a normative environment that rejects GBV in all its forms.

## Introduction

Gender broadly reflects a multidimensional relational construct that describes attributes, roles, behaviors, and norms typically characterized within a spectrum of feminine and masculine, while also speaking to power processes between and among individuals within health and social systems [1,2]. Gender inequities faced by women and girls globally manifest divergently in health risks, behaviors, and health care access, privileging those with greater positional power within their social identities, material resources, and social capital. Overcoming gender inequities in health is a crosscutting principle underlying the sustainable development goals (SDGs), including SDGs 3 and 5, which speak to ensuring healthy lives and well-being for all and achieving gender equality and empowering all women and girls, respectively [1]. Gender-based violence (GBV) refers to physical, psychological, and sexual harassment or abuse toward women and girls (and sexual and gender minorities) perpetrated by men. GBV provides an example of how gender inequities manifest in and affect the health and well-being of those who present as having an “inferior” positional status, including women and sexual and gender minorities.

GBV is pervasive with 27% of married women of reproductive age globally, and 33% in sub-Saharan Africa reporting a lifetime prevalence of physical or sexual intimate partner violence. Global estimates among young people aged 15-24 years (ranging 15%-30%) demonstrate that GBV occurs early in the life course [3]. GBV has been seen as an extension of misogyny—hatred, contempt for, or prejudice toward women that can be expressed directly and indirectly through negative stigma toward women, discriminatory health care systems, sexual and reproductive rights deprivation, and community-specific expressions and norms that belittle, defame, or slander women [4]. Sexual consent education, defined as the active agreement and communication of willingness to engage in sexual activity, is a crucial element for prevention of GBV [5,6]. While subtle forms of GBV such as gender-discriminatory and inequity-perpetuating perspectives were previously less recognized, recent studies increasingly draw attention to wider group-based trends in anti-women or anti–gender-equitable rhetoric through assessed conversations in the virtual space—web-based documentation, social media, applications, and digital platforms [7-9]. Women’s vulnerability to GBV by stalking and monitoring their movement without consent increases with the use of digital tools [7]. The United Nations Broadband Commission report raised the alarm of web-perpetuated GBV in the form of verbal harassment and threats toward real-life violence, which poses an increased concern for young women between 18 and 24 years of age globally and in low- and middle-income settings specifically [10].

Currently under debate is women’s exposure to web-based slut-shaming—stigmatization in the form of insults based on actual or one’s perceived sexuality and behaviors—which can lead to experiencing sexual double standards and negative psychological health [11,12]. While exposure to slut-shaming via texting and some web-based platforms may reflect social norms that penalize girls more than boys for any type of real or perceived sexual or sexualized activity [11], slut-shaming on public-facing platforms like Facebook may also result in a reverse double standard where a male who shames a female is judged harshly [12].

In the Nigerian context, gender inequities stem from household and societal level behavioral expectations of women and men, including male dominance or female subservience, male strength or female weakness or ignorance, patriarchal norms, and religious or customary privileging of men over women, which are further reinforced by political indifference and lack of protective policy [13,14]. Collectively, these dynamics affect embodied gendered relationships and often lead to various inequitable health outcomes such as risky sexual behaviors among young people enacting learned stereotypes [15], alcohol use–induced shaming of women [16], limited access to skilled prenatal and delivery health care [17], and GBV generally and in and around pregnancy [13,18]. Despite the pervasiveness of gender inequalities and GBV globally and in Nigeria, Nigerians have historically maintained a culture of silence and underreported instances of GBV, which hinders its tracking [14]. A study conducted among internally displaced women in a southern Nigerian state revealed that being an adolescent, unmarried, and of Hausa descent made women more vulnerable to GBV [19]. Sexual violence has also been reported to be high (47.1%) among young primary school students, while physical, sexual, and emotional GBV was estimated to have a prevalence of nearly 60% among those attending a tertiary educational institution in northern Nigeria [20,21]. To strengthen reporting, the Nigerian government, in partnership with the European Union, launched the GBV data situation room and dashboard to promote web-based reporting in 2022 [22].

Similar to increasing global trends in social media use, the Nigerian population is increasingly engaging on a variety of digital platforms through their smartphones. According to Statista’s global consumer survey, social media users in Nigeria have increased from 18 million in 2017 to around 34 million in 2022, with approximately 58% of users being men [23]. As of late 2021, WhatsApp (Meta Platforms, Inc) was the most used platform (approximately 92%), followed by Facebook (approximately 86%, Meta Platforms, Inc), Instagram (approximately 78%, Meta Platforms, Inc), and Facebook Messenger (approximately 71%, Meta Platforms, Inc) [24]. Given the increasing exposure and influence of smartphones, social listening—namely cataloguing and counting content and observing topical trends in digital platform–based conversations—has gained traction for a variety of health topics including gender broadly and GBV in particular [7,9,25,26]. This approach, which has been applied in Nigeria and other African settings, allows for researchers to monitor and evaluate web-based audiences’ opinions and behaviors while offering practitioners valuable insights into possible ways to effect social and behavior change [26].

The aim of this paper is to explore how gender-related internet-based conversations in Nigeria, specifically those related to sexual consent and slut-shaming, manifest themselves and whether they have changed in the last 5 years. Additionally, we explore what role events or social movements have in shaping gender-related narratives in Nigeria.

## Methods

### Study Design

This study uses social listening techniques to retrospectively conduct quantitative and qualitative content analysis of internet-based data related to GBV in Nigeria between April 2017 and May 2022, specifically focusing on the topics of sexual consent and slut-shaming.

### Data Sources

Two types of data sources were used: public social media posts (n=12,031) including forums and blogs, and private search data (n=1,956,213 individual searches). Data were sourced using English, Hausa, Yoruba, and Igbo search terms, and all data points were translated into English for analysis using proprietary machine-powered artificial intelligence (AI). For quality assurance purposes, translations were checked with Google Translate to ensure alignment, consistency, and accuracy.

Social media data consisted of posts from Twitter (n=10,226, 85%), Facebook (n=481, 4%), YouTube (n=362, 3%), Reddit (n=120, 1%), forums (n=481, 4%), and blogs (n=361, 3%), and reflect the audiences’ thoughts, attitudes, and opinions. The sources of data for this study do not match the most used social media platforms in Nigeria due to privacy restrictions implemented by the 4 most popular platforms’ parent company. Among the Twitter-using population in Nigeria who report using the platform daily or more than once a day, 58.6% are male, 70.5% are between 16 and 34 years of age, and 46.3% are single [27].

Private internet search data consist overwhelmingly of Google searches (n=1,924,914, 98.4%), with additions from Bing (n=27,582, 1.41%; Microsoft Corp) and Yahoo (n=3717, 0.19%), and enable us to see what searches are conducted with relation to a specific topic at a given time. These data provide insight into what topics an audience is trying to understand or educate themselves on using search engines.

### Procedures

The study was conducted in three phases: (1) an extraction of data related to several broad gender-related key terms, (2) a focused analysis of select thematic clusters, including sentiment and emotion detection, and (3) a time trend analysis of topic-specific conversation volume and search data.

For the data extraction, we curated a list of relevant, high-volume keywords (see Multimedia Appendix 1) to then extract search volume to calculate topic narrative size (percentage of total number of searches assigned to a topic) and growth between April 2017 and May 2022.

We leveraged proprietary AI to identify and cluster data points (ie, individual social media posts or search queries) related to gender norms discourse in Nigeria. This model uses a density-based clustering algorithm that positions data points that show overlap into a strongly defined cluster, eliminating data noise (ie, irrelevant posts) in between. Our proprietary semiotics AI model—a model that processes natural text and assigns meaning to it based on observations it makes—generated 14 initial clusters of conversation, including discourse in posts (n=12,031) made by organizational (n=1684, 14%) and individual (n=10,347, 86%) social media accounts. After identifying the most engaging conversations, using the number of post retweets as proxy, the following 7 clusters emerged: bodily autonomy, GBV, gender equality, women and sexuality, maintaining traditional gender roles, mistrust of women, and money matters (see Multimedia Appendix 1 for a descriptive table of all clusters). This paper presents findings from 2 subtopics in 2 clusters, selected by their prominence within their cluster as well as relevance for GBV and public health prevention initiatives, such as comprehensive sex education: (1) the subtopic of sexual consent within the GBV cluster and (2) the subtopic of slut-shaming within the cluster of female sexuality. We chose to analyze these 2 subtopics together, as slut-shaming narrative is often used to explain or justify nonconsensual sexual behavior in social narratives [28]. There were no differences in the data sources between the chosen subtopics.

**Figure 1:**
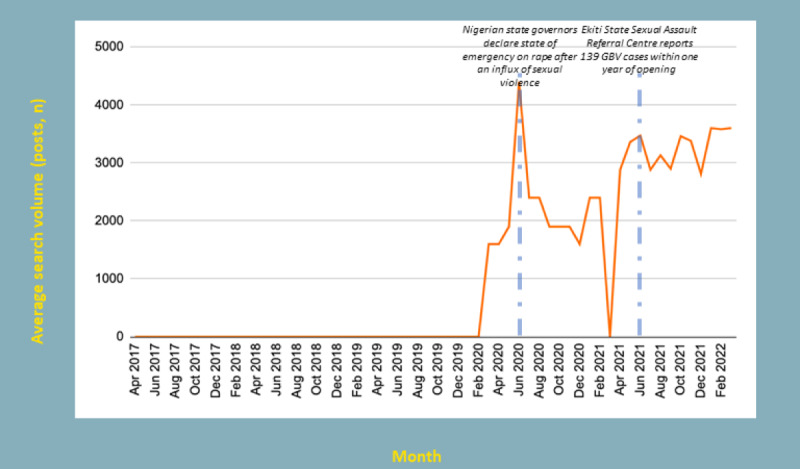
Search data (Google, Yahoo, and Bing) related to sexual consent. GBV: gender-based violence.

**Figure 2:**
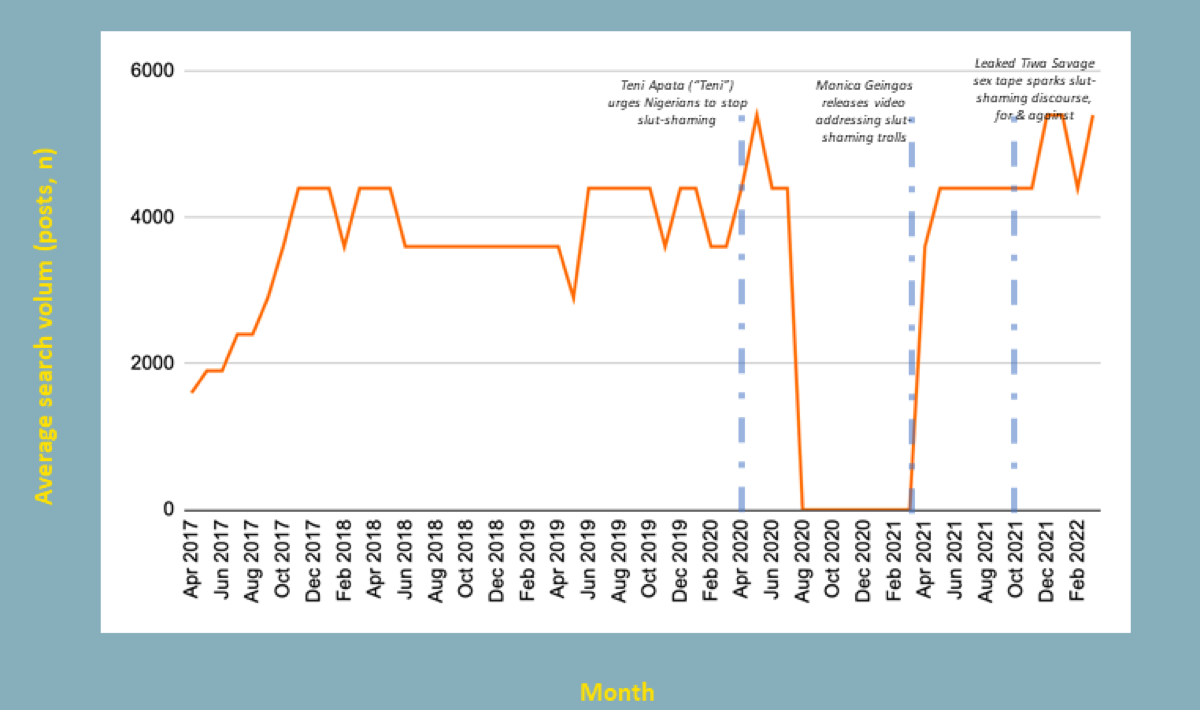
Search data (Google, Yahoo, and Bing) related to slut-shaming.

We calculate the rate of change in subtopic specific conversation volume using the following formula: rate of change = [((average monthly volume end-average monthly volume start)/average monthly volume end) x 100]. We used Facebook Audience Insights, a Facebook analytical tool, to aggregate information and determine the dominant sex and age of searchers within each narrative. We then extracted and visualized social media post volume over the past 5 years to contrast with key event timelines (see Table 1), using Twitter searches’ geocode function to ensure that all data were limited to Nigeria. Qualitative trend analysis was conducted by thematically analyzing social media posts within the 2 selected cluster subtopics by publication year. To assess correlation between key events and changes in internet-based posts and searches, we compared premonthly and postmonthly volumes using chi-square tests (illustrated in Figure 1 and Figure 2).

Subsequently, we used emotion and sentiment models developed through collaboration efforts led by the Robert Koch Institute that are trained to recognize and predict patterns in a given text to determine dominant discourse attitudes, such as positivity, negativity, joy, anger, optimism, or sadness [29]. Textual data points can exhibit multiple emotions and can be categorized as such. Hence, emotion-related results within each subtopic may not add up to 100%. We present examples of social media posts representing these emotions to illustrate the breadth of internet-based discourse under the selected cluster subtopics.

**Table 1:** Key events explored in relation to gender equity and gender-based violence.

Social media hashtag	Description of event or movements	Date of initiation (month of analysis)
#ArewaMeToo	A localized version of #MeToo—a global movement founded in 2006 by author Tarana Burke and popularized on Twitter in October 2017—started by entrepreneur and development worker Fakhriyyah Hashim. Arewa is a Hausa term for the northern part of Nigeria.	February 3, 2019 (February 2019)
#ChurchToo	A derivation of #MeToo, this movement exposes sexual abuse and harassment perpetrated in church and religious settings.	July 1, 2019 (July 2019)
#SexforGrades	“Sex for Grades” is a BBC documentary uncovering sexual harassment at the University of Lagos, Nigeria, and the University of Ghana.	October 7, 2019 (October 2019)
Big Brother Naija (#BBNaija)	A Nigerian reality competition TV series which occurs annually and is based on the global Big Brother television franchise, in which contestants live in an isolated house and compete for a large cash prize and other material prizes. This show was chosen due to its relevance in popular culture and its implicit and explicit sexual content.	July 19, 2020 (July 2019)

### Ethical Considerations

Analyses presented in this paper were granted an institutional review board exemption by the Population Council research ethics committee due to the public nature of the internet-based data used.

## Results

### Data Description

Internet-based conversation about sexual consent has grown an astonishing 72,633%, from an average of 3 posts to an average of 2182, while slut-shaming conversation (perpetrating or condemning) has shrunk by 9% from an average of 3560 posts to an average of 3253 in the last 5 years (see Table 2). Despite the enormous growth of internet posts related to sexual consent, conversation volume around slut-shaming at the end of the study period remained higher than that of sexual consent.

Table 3 shows that for sexual consent and slut-shaming, the majority of people posting were located in southwest Nigeria (where Lagos City is located), followed by north-central Nigeria (which includes the Federal Capital Territory). The distribution of topic-specific posts is not consistent with the distribution of the population with access to the internet in the country, which ranges from 53% to 68% across geopolitical regions [30]. Noteworthy is the difference in engagement across topics among those in the northeast and northwest regions, posting more about sexual consent than slut-shaming.

**Table 2:** Topic-specific web-based conversation volume and rate of change by start and end date.

Topic	Posts at start (average in April 2017), n	Posts at end (average in May 2022), n	Total data points, n	Rate of change (2017-2022), %
Sexual consent	3	2182	65,450	72,633
Slut-shaming	3560	3253	204,400	−9

**Table 3:** Topic-specific conversation prevalence by region.

Region	Gender-based violence (sexual consent; n=65,450), n (%)	Women and sexuality (slut-shaming; n=204,400), n (%)
Southwest	37,961 (58)	145,124 (71)
North-central	14,399 (22)	42,924 (21)
South-south	5236 (8)	10,220 (5)
Northwest	4582 (7)	2044 (1)
Southeast	1963 (3)	4088 (2)
Northeast	1309 (2)	0 (0)

### Thematic Analysis

#### GBV and Sexual Consent

Conversations on the topic of GBV consisted of posts related to awareness raising (ie, statistics and testimonies), specific advocacy (eg, domestic violence and street harassment), harm reduction education (ie, sexual consent), and societal expectations around gender roles.

#Before April is over, don’t forget it’s sexual assault awareness month, No means No! It doesn’t matter what you wear it doesn’t matter who you are, male or female. For anyone going through this, the shame is not yours to carry...it’s the monsters who do this!

Male

Testimonies also took on the role of an accountability mechanism, with posters tagging businesses to make them aware of behavior associated with their employees or representatives.

So I was sexually harassed by a @Boltapp_ng [ride sharing app] rider yesterday. It made me too scared to leave the house because the guy said “I know your area” and “you’re lucky you’re a good girl” when he dropped me off. This was after he went into details of how he’ll have sex with me for hours.

Female

Advocacy-related content highlighted the incongruence between focusing prevention efforts on those most powerless rather than those perpetrating violence and exerting power.

The problem with groups who deal with rape is that they try to educate women about how to defend themselves. What really needs to be done is teaching men not to rape. Go to the source and start there. #sexualassault #metoo #rape #sexualabuse #consent #survivor #sexualharassment.

Female

Other posts tried to trace the lines connecting topics such as the objectification of female bodies and the importance of consent.

If you’re shocked that 11 thousand men are in a group where they share nudes of women then you haven’t been listening. Men show and tell us EVERYDAY that they don’t care about consent and women’s autonomy. Most importantly, they view women solely as sexual objects.

Female

Analyzing posts discussing sexual consent as an effort to prevent instances of sexual assault, violence, and coercion, we see posts in response to rising rates of sexual violence and harassment.

For many social media posters, the elements of consent must be made explicit in an environment where consent does not seem to be intuitively understood. Both female and male social media users introduce or define elements constituting consent such as age; sobriety; and informed, continual, active consent. Posts also offer victims of GBV support. Global campaigns like “#nomeansno” unburden the survivor of responsibility in instances of sexual violence.

Rules of sexual consent:

Ask every time

The best way to get sexual consent is to ask politely

Don’t jump into conclusions before jumping to bed

Consent is to be given at the beginning of the process and at every stage of the sexual process

#SexualConsentwithBenee.

Female

Women appearing to smile whilst being sexually harrassed does not mean they are okay with it. Smiling is either a de-escalating technique or an awkward response. NOT to be confused with consent.

Female

#### Female Sexuality and Slut-Shaming

The second conversation cluster analyzed was related to Nigerian women embracing or acknowledging their sexuality, and the backlash that can generate.

Ahu and Karo were the first sex positive women I ever met in my whole life. It’s part of why I love them so much. Because I felt normal and that my desires weren’t devilish.

Female

While women are creating space for sexual empowerment, alternate posts weaponize this discourse to punish, shame, or dehumanize women for their sexuality and sexual freeness (real or imagined) as women become increasingly vocal in web-based spaces about their sexuality. While men are the drivers of this narrative, women are not exempt from also shaming women for their sexuality or dress. However, women do almost exclusively make up discourse criticizing slut-shaming, whereas men in that respect are largely absent. The following are posts defying the gendered expectation of men perpetrating slut-shaming and women criticizing it.

This is the reason why women are objectified and then, you’ll come out to lash out at men when they say all kinds of things about women being sex objects. Cover up madam!! All of these on social media is totally unnecessary. F’asobora [meaning “cover yourself with cloth” in Yoruba language]!

Female

As for those saying “she posted it herself” that’s no excuse for slut shaming; the fact that you left the millions of other pictures of her & specifically went for the one that fits your fucked up moral depiction of a loose woman, says a lot that you think she deserves Blackmail.

Male

Notably, the slut-shaming narrative is receding. A myriad of public discourse (Teni Apata—an influential Nigerian musician—publicly condemned slut-shaming in 2020, and Monica Geingos—the first lady of Namibia—also spoke out about her own experiences) may have deterred users from perpetuating slut-shaming culture in web-based spaces.

Finally, we also find posts using humor to highlight inconsistencies in prevalent discourse related to women using sex and their bodies to gain access to privileged spaces, while also noting that women rarely have access to privileged spaces.

Lmao serious wahala for women. Small thing, you’ve slept your way to the top [of an organizational hierarchy]. When you go and check the top, there are hardly any women there [crying face emoji, crying face emoji]. So really, which way?

Female

### Time Trend Analysis

#### GBV: Sexual Consent

Through qualitative analysis of web-based discourse over the past 5 years, a majority of web-based users are increasingly defining what consent means, culturally and legislatively. Ambiguity around certain elements of consent—like the legal age of consent—dominated web-based discourse in earlier years, particularly due to the complexities of Nigeria’s criminal justice system, which handles legal age of consent differently under the criminal and penal code (see Table 4). Traditional practice under the penal code, based on Sharia law, enables many to believe consent can exist beginning at 11 or 12. Later posts continue to show lingering confusion around the country’s official age of consent, yet most users are counteracting what are perceived as misinformed posts.

**Table 4:** Qualitative time trend for topic of sexual consent.

Year	2019	2020	2021
Illustrative quote	“Why isn’t the legal consent age of Nigeria Crystal clear?? Is it 12 or 18?? How are we even discussing this in 2018??” [Female]	“Child sexual abuse in Nigeria is an offence under several sections of chapter 21 of the country’s criminal code. The age of consent is 18. UNICEF reported in 2015 that 1 in 4 girls and 1 in 10 boys in Nigeria had experienced sexual violence before the age of 18.” [Female]	“The legal age of consent in Nigeria is 18. Some sites erroneously report 11, but the law prohibits sexual intercourse with children between the ages of 11, 12, 15, to 18 years, with possible life imprisonment. Knowledge is power.” [Female]

Longitudinal analysis of search data for sexual consent shows greater interest in mid-2020 that coincides with Nigerian state governors’ declaration of a state of emergency on rape via the Nigerian Governors Forum (see Figure 1).

Other events of interest also coincide with spikes in social media conversation volume, such as #ChurchToo, #SexforGrades, and #BBNaija (data not shown). #ChurchToo located the consent conversation within religious spaces, specifically triggering discourse around skewed power dynamics and their impact on coercion after a popular Nigerian singer publicly accused a pastor and head of a large church in Abuja of raping her as a teenager. Meanwhile, #SexForGrades invited consent-based discourse as Professor Gyampo—the subject of the undercover documentary—asked his “student” if she had been “kissed violently,” sparking greater conversation around consent and sexual violence. Finally, in season 5 of Big Brother Naija, a contestant named Laycon made waves by rapping about consent and GBV. This message was reshared and circulated by many Nigerian users, celebrating his vocality and the effect this may have on educating the masses on consent.

#### Female Sexuality: Slut-Shaming

Slut-shaming is receding in terms of number of posts. Since 2017, female users are increasingly standing up to slut-shamers in web-based spaces in an effort to stigmatize, rather than internalize, judgements around female sexuality. While resignation or acceptance are more common in earlier discourse, posts are increasingly returning the very shame that aims to silence them (see Table 5). In this same timeframe, negative discourse receives 47% (167/355) of the narrative’s total tweet replies, compared to neutral (92/355, 26%) or positive (96/355, 27%) posts (data not shown).

**Table 5:** Qualitative time trend for topic of slut-shaming.

Year	2018	2019	2021
Illustrative quote	“If we start the #MeToo movement in Nigeria..., most importantly, they will turn the tables and slut shame. Women can never win in this part, our men are illiterates. A luta Continua [strong arm emoji].” [Female]	“In 2019 a Nigeria lady will upload a picture of herself wearing a bikini and you’ll slut shame her. You’ll go to foreign countries and see women walking all around in bikini and say God lives here. Double standard will kill a lot of you no be curse.” [Female]	“If you have a mother and also have a sister, and you slut-shame hardworking women, shame on you. And if you are a woman and partake in such stupidity, I don’t have words for you. You all should know better.” [Male]

Longitudinal analysis of search data shows correlation between macro influencer Tweets and search activity related to this topic (see Figure 2).

#ArewaMeToo sparked discourse from both sides of the slut-shaming discussion: for some users, it proved to be an opportunity to further shame women for their sexual experiences such as assault or harassment, despite the violence involved in them. Others pushed back, urging women to ignore their shamers. Big Brother Naija, season 5 also depicted and inspired slut-shaming and included examples of women shaming each other which invited discourse on how men are not always the perpetrators. #ChurchToo resulted in protests against the church as an anti-woman establishment perpetuating rape culture and shaming activists for speaking out. Thus, the movement caused an increase in discourse around slut-shaming at large.

### Emotion Analysis

Emotion analysis shows that a majority of discourse surrounding sexual consent (n=1682) is angry (n=1213, 73%) and negative (n=1127, 67%), particularly in June 2020, with 6% (n=102) of sexual consent narrative being sad, 10% (n=166) being optimistic, and 6% (n=102) being joyful. For the topic of slut-shaming, 64% (n=226/355) of all posts contain anger, while only 5% (16/355) of posts speak of slut-shaming optimistically, or in compassionate terms. Slut-shaming posts are virtually all negative—whether it be from users condemning or perpetuating slut-shaming. Table 6 shows example posts of discourse detected within each emotion category.

**Table 6:** Emotion analysis with representative posts.

Emotion	Sexual consent		Slut-shaming	
	Posts (n=1682), n (%)	Example post	Posts (n=355), n (%)	Example post
Inspiration	101 (6)	“This is one of the most important movies you can ever see on Rape, Molestation & Consent. It makes it very clear how NO means NO. Yo, I cried! This is a well done movie. Thank you @SrBachchan Seen “Pink” on Netflix yet?” [Male]	67 (19)	“Kim Kardashian has being relevant for more than a decade and she’s still doing good for herself and her family. For someone people slut-shame, insults and hates and is always called ‘talentless’ she’s doing super amazing 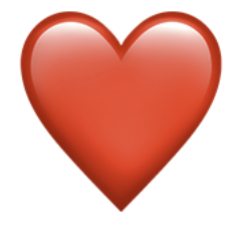 I love her...she inspires me.” [Female]
Optimism or compassion	166 (10)	“SAY NO TO RAPE 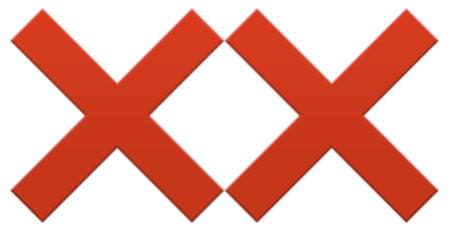 Speaking from a concerned heart, Let’s stop all the violence and start to spread love. Together we can build a better country. #justiceforuwa #justiceforbarakat #justicefortina #justiceforuwaandtina.” [Male]	16 (5)	“Hookup girls or Ashewo at large, they are normal human beings like everyone else. Their kind of lifestyle or work doesn’t make them any less. They are far harmless than people care to know. Give them a chance.” [Male]
Anger	1213 (73)	“I still don’t understand this “Say no to Rape/Rapists” statement, okay we’ve said no, what’s next? Is justice actually being served? Or everything is just okoto [nonsense in keeping with the status quo].” [Female]	226 (64)	“omooo y’all prefer slut shaming a victim to holding a rapist accountable.” [Female]
Sadness	102 (6)	“Tales of pain, thoughts that sting Breathes with me still. A helpless situation. I strolled the path of weakness, the beasts gripped and tore at me, Fought to nought, Overpowered, they had their ways, Took turns, they forced themselves on me Oh the PAIN! #saynotorape.” [Female]	33 (9)	“#BBNaijaReunion I saw low self esteem 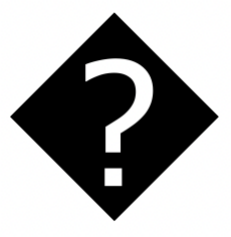 the ladies going at each other and slut shaming themselves on national TV...see wetin our children don reduce to for fame & fortune...o ma shey.” [Male]

## Discussion

Internet-based conversation about sexual consent has grown in the last 5 years, fueled by organic social movements such as #MeToo, #ArewaMeToo, and mass media stories such as #SexforGrades, while slut-shaming conversation (both perpetrating and condemning) has shrunk. Thematic analysis shows conversation revolving around the objectification of women, poor comprehension of elements of consent, and advocacy efforts to educate the public about consent. On the other hand, people posting content on the internet are also creating space for sexual empowerment and expressions of sex-positivity, pushing back against others who weaponize these posts in support of slut-shaming narrative. Time trend analysis shows that social movements and popular culture events such as #ArewaMeToo and #ChurchToo movements, the #SexforGrades scandal, and the #BBNaija program can trigger significant growth in web-based narratives, and a greater sense of empowerment in advocating for education around the age of consent and rejecting slut-shaming. Emotion analysis illustrates that the majority of social media narratives for both sexual consent and slut-shaming reflect anger.

The Nigerian legal, religious, and social context is complex and has important implications for combatting GBV. Specifically, Nigeria’s criminal justice system is governed by 2 separate sets of legislation known as the penal code and the criminal code. The minimal age at which a person can participate in sexual activity, often known as the legal age of consent, is handled differently in these two codes. The criminal code, which is applicable to the southern states of Nigeria, is based on British common law, while the penal code, which is applicable to the northern states of Nigeria, is based on Islamic (sharia) law. While the criminal code sets the age of consent at 18, the penal code has no clear-cut age of consent. Sexual consent under the penal code can only be recognized under marital union. This means a girl at 12 years of age could be considered an adult, if in a marital union with a male spouse. The two laws’ divergent legal consent ages reflect Nigeria’s diverse cultural and religious landscape. The penal code of the primarily Muslim northern states reflects the community’s cultural and religious beliefs, which views sexual consent as legitimate only in marriage. The majority-Christian southern states, on the other hand, have opted for the international minimum age, which is 18. It is crucial to note that having sex with a minor is illegal regardless of the age of consent established by these codes. Both the penal code and the criminal code make having sex with a minor a crime, and they both impose harsh punishments on offenders [31,32]. Given this variation in state laws, as well as cultural and religious practices, social listening can be a useful tool to track and understand the changing sentiments toward GBV across specific regions or groups in Nigeria.

Programmatic implications stemming from this work include leveraging popular cultural events and social movements with internet-based presence to elevate transformative messaging that supports gender equity. This may include the use of hashtags to link public health and gender equity–related campaigns and content to current events or social movements, as well as the use of “micro” influencers who have potential for reaching specific audiences to deliver targeted health related information and elicit positive responses [33]. Furthermore, public health stakeholders should be prepared to capitalize on changes in social media discourse, getting out in front of GBV or gender equity or inequity conversations to help shape them and provide links to trusted resources and services. Public health practitioners and stakeholders must also leverage trusted organizations and internet influencers to drive interest and attention to improve GBV reporting and fight misinformation that fuels negative gender norms and promotes understanding around consent. Further research should investigate the relevance and effectiveness of micro influencers for challenging unhealthy social norms surrounding GBV. Finally, there is insufficient evidence currently on the effectiveness of behavior change theories in social media−based and digital interventions, which should be an area of further research [34].

There are several limitations inherent to social listening, particularly in settings where there is nonrandom access to social media and mobile technology, as is the case in Nigeria. Inequities in access to these technologies lead to lack of generalizability [26]. Furthermore, inequities in access to the internet, mobile phones, and social media leads to sociodemographic skews in the population that participates in web-based and digital conversation of any nature. In the case of Nigeria, the gender gap has been narrowing in the past 5 years. In 2022, 55% of social media users were male and 45% were female [27]. Almost half of social media users are younger than 24 years. Additionally, social listening reflects only the narratives and discourse happening in public, internet-based spaces, the majority coming from Twitter, which has less restricted access to data contained within its platform. Data analyzed may not mirror narratives expressed in other platforms, as Twitter-using audiences may differ significantly from those who use other more popular platforms, such as Facebook, or non–internet-based channels. Finally, our analyses reflect thematic trends from 2017 to 2022 based on a list of social listening key search terms (see Multimedia Appendix 1). Although the list is comprehensive, we could have missed relevant language or conversations that extend beyond the terms used.

Despite these limitations, social listening methodology can bring valuable insights to understand evolving trends in health-related narratives, including those related to gender-related discourse. Public health practitioners, researchers, and other stakeholders, including policymakers, government officials, and social advocates, should be prepared to capitalize on social media events and organic social movements to help shape the conversation in support of a normative environment that rejects GBV in all its forms.

## Appendix

Multimedia Appendix 1 Key search terms and initial thematic clusters.

## Abbreviations

AI: artificial intelligence
GBV: gender-based violence
SDG: sustainable development goal

## Data Availability

The data set generated and analyzed during this study is available from the corresponding author on reasonable request.

## References

[1] Manandhar M, Hawkes S, Buse K, Nosrati E, Magar V (2018). Gender, health and the 2030 agenda for sustainable development. Bull World Health Organ.

[2] Connell R (2012). Gender, health and theory: conceptualizing the issue, in local and world perspective. Soc Sci Med.

[3] (2021). Violence against women prevalence estimates, 2018. World Health Organization.

[4] Arsawati NNJ, Bunga D (2022). Misogyy as violence in gender perspective. Int J Bus Econ Soc Dev.

[5] Eze UO (2013). Prevention of sexual assault in Nigeria. Ann Ib Postgrad Med.

[6] Burton O, Rawstorne P, Watchirs-Smith L, Nathan S, Carter A (2023). Teaching sexual consent to young people in education settings: a narrative systematic review. Sex Educ.

[7] Afrouz R (2023). The nature, patterns and consequences of technology-facilitated domestic abuse: a scoping review. Trauma Violence Abuse.

[8] Usher K, Durkin J, Martin S, Vanderslott S, Vindrola-Padros C, Usher L, Jackson D (2021). Public sentiment and discourse on domestic violence during the COVID-19 pandemic in Australia: analysis of social media posts. J Med Internet Res.

[9] Laskovtsov A (2020). Navigating the manosphere: an examination of the incel movements' attitudes of sexual aggression and violence against women [thesis]. Eastern Kentucky University.

[10] (2015). Urgent action needed to combat online violence against women and girls, says new UN report. UN Women.

[11] Goblet M, Glowacz F (2021). Slut shaming in adolescence: a violence against girls and its impact on their health. Int J Environ Res Public Health.

[12] Papp LJ, Hagerman C, Gnoleba MA, Erchull MJ, Liss M, Miles-McLean H, Robertson CM (2015). Exploring perceptions of slut-shaming on Facebook: evidence for a reverse sexual double standard. Gender Issues.

[13] Gage AJ, Thomas NJ (2017). Women's work, gender roles, and intimate partner violence in Nigeria. Arch Sex Behav.

[14] Abayomi AA, Olabode KT (2013). Domestic violence and death: women as endangered gender in Nigeria. Am J Sociol Res.

[15] Dumbili EW (2016). Gendered sexual uses of alcohol and associated risks: a qualitative study of Nigerian University students. BMC Public Health.

[16] Ikuesan BA (1994). Drinking problems and the position of women in Nigeria. Addiction.

[17] Yaya S, Okonofua F, Ntoimo L, Udenige O, Bishwajit G (2019). Gender inequity as a barrier to women's access to skilled pregnancy care in rural Nigeria: a qualitative study. Int Health.

[18] Andersson N, Omer K, Caldwell D, Dambam MM, Maikudi AY, Effiong B, Ikpi E, Udofia E, Khan A, Ansari U, Ansari N, Hamel C (2011). Male responsibility and maternal morbidity: a cross-sectional study in two Nigerian states. BMC Health Serv Res.

[19] Adejumo OA, Ntoimo L, Odimayo MS, Adebimpe WO, Okiei B, Osungbemiro W, Olajuyigbe E, Igbafe K, Temitayo-Oboh A, Faboya T, Oludiran O, Okonofua FE (2022). Experience of gender-based violence by internally displaced women in Southern Nigeria: a cross-sectional study. J Interpers Violence.

[20] Iliyasu Z, Abubakar IS, Aliyu MH, Galadanci HS, Salihu HM (2011). Prevalence and correlates of gender-based violence among female university students in Northern Nigeria. Afr J Reprod Health.

[21] Ekine A (2020). Gender-based violence in primary schools: Nigeria. Center for Universal Education at Brookings.

[22] National gender based violence dashboard. Federal Ministry of Women Affairs Nigeria.

[23] Most used social media platforms in Nigeria as of the 3rd quarter of 2022. Statista.

[24] (2022). Social media usage by platform type in Nigeria in 2022. Statista.

[25] Kavanagh E, Litchfield C, Osborne J (2019). Sporting women and social media: sexualization, misogyny, and gender-based violence in online spaces. Int J Sport Commun.

[26] Silva M, Walker J, Portillo E, Dougherty L (2022). Strengthening the Merci Mon Héros campaign through adaptive management: application of social listening methodology. JMIR Public Health Surveill.

[27] (2022). Homepage. Global Web Index.

[28] Aborisade RA (2023). On the ‘darkness of dark figure’ of sexual crimes: survivors' rape reporting experiences with the Nigerian police. Int J Law Crime Justice.

[29] Chinea-Rios M, Franco-Salvador M, Benajiba Y (2020). Aspect on: an interactive solution for post-editing the aspect extraction based on online learning. https://aclanthology.org/2020.lrec-1.612/.

[30] (2019). Social media poll report. NOIPolls.

[31] Singh JA, Jogee F (2018). Age of consent: legal, ethical, cultural and social review: Nigeria country report. Southern African AIDS Trust.

[32] (2021). Nigeria 2021 human rights report. United States Department of State: Bureau of Democracy, Human Rights and Labor.

[33] Bonnevie E, Smith SM, Kummeth C, Goldbarg J, Smyser J (2021). Social media influencers can be used to deliver positive information about the flu vaccine: findings from a multi-year study. Health Educ Res.

[34] Castle S, Silva M (2019). Family planning and youth in West Africa: mass media, digital media, and social and behavior change communication strategies. Population Council.

