# Supplementary material for: Gender-Based Violence Narratives in Internet-Based Conversations in Nigeria: Social Listening Study
Source: J Med Internet Res. 2023 Sep 15;25:e46814. doi: 10.2196/46814 (PMC10541644; doi:10.2196/46814)
Supplement: Multimedia Appendix 1 [file jmir_v25i1e46814_app1.docx]

**1. Key search terms:**

- - Sexual behavior
    - One night stand
    - Sex before marriage
    - Casual sex
  - Contraception
    - Family planning
    - Condoms
    - Pill
    - Diaphragm
    - Contraceptive implant
    - Contraceptive injection
    - Contraceptive patch
    - Female condoms
    - IUD
  - Sexism & Gender
    - Gender identity
    - Male
    - Female
    - Non-binary
    - Gender fluid
    - Trans
    - Transexual
    - International day of the girl child
    - International women’s day
  - Menstruation / Puberty
    - Puberty
    - Menstruation
    - Period
  - Relationships
    - Boyfriend
    - Girlfriend
    - Partner
    - Husband
    - Wife
    - Nigerian slang for 'sugar daddy/side dish'
    - Is there a female version of sugar daddy?
  - STIs
    - Chlamydia
    - Genital Herpes
    - Gonorrhea
    - HIV/AIDS
    - HPV
  - Sexual violence
    - Abuse / abusive
    - Abused
    - Rape
    - Sexually violent
    - Sexually vulnerable
    - Consent
    - GBV
    - Misogyny
  - Health
    - Illness
    - Maternal health
    - Antenatal health
  - Agency
    - Power
    - Capable
    - Ability
    - Victim
    - Decision
    - #metoo
    - #churchtoo
    - #ArewaMeToo
    - Discrimination
    - Women’s rights
    - Gender equality
    - #girlpower
    - Intersectionality
  - Norms
    - Men should
    - Women should
  - Autonomy
    - Feminism / Feminist
    - Patriarchy

1. **Initial cluster extraction / distribution**

| *Cluster* | *Narrative* | Total Search | **% Share** |
| --- | --- | --- | --- |
| Bodily Autonomy/Adversity | Menstruation | 31690 | 1.620% |
|  | **Women's Health Awareness** | **442603** | **22.626%** |
| Bodily Autonomy/Adversity Total |  | 474293 | 24.245% |
| Gender Based Violence | Addressing Marital/Domestic Violence | 73270 | 3.746% |
|  | Collective Female Experience | 49070 | 2.508% |
|  | Genital Mutilation Awareness | 49430 | 2.527% |
|  | Harassment in Public Spaces | 25500 | 1.304% |
|  | Holding Politicians Accountable | 100 | 0.005% |
|  | **Sexual Consent** | **65450** | **3.346%** |
|  | Women Without Agency | 8230 | 0.421% |
| Gender Based Violence Total |  | 271050 | 13.856% |
| Maintaining Traditional Gender Roles | Men As Leaders | 600 | 0.031% |
|  | Men as Protectors | 350 | 0.018% |
|  | **Reclaiming Men's Rights** | **18820** | **0.962%** |
| Maintaining Traditional Gender Roles Total |  | 19770 | 1.011% |
| Mistrust of Women | **Capacity for Cheating** | **170760** | **8.729%** |
|  | False Accusations | 3670 | 0.2% |
| Mistrust of Women Total |  | 174670 | 8.929% |
| Money Matters | **Female Financial Exploitation** | **116790** | **5.970%** |
|  | Female Financial Reliance | 280 | 0.014% |
|  | Men as Providers | 50 | 0.003% |
|  | Resisting Stereotypes | 180 | 0.009% |
| Money Matters Total |  | 117300 | 5.996% |
| New Age Gender Equality | Engaging Male Allies | 640 | 0.033% |
|  | Financial Opportunity | 9770 | 0.499% |
|  | Rapid Reform | 5200 | 0.266% |
|  | **The Importance of Women's Rights** | **132110** | **6.753%** |
| New Age Gender Equality Total |  | 147720 | 7.551% |
| Women & Sexuality | Against Premarital Sex | 10370 | 0.530% |
|  | Male Entitlement | 90 | 0.005% |
|  | Male Ownership/Possessiveness | 3130 | 0.160% |
|  | Resisting Objectification | 640 | 0.033% |
|  | **Slut Shaming** | **204400** | **10.449%** |
| Women & Sexuality Total |  | 218630 | 11.176% |
| **Total of Narratives Analyzed** |  | **1150933** | **58.84%** |
